# A multi-cytokine inhibitory peptide (BNZ 132-1) that is a potential therapeutic agent for HAMTSP and other necrotizing diseases.

**DOI:** 10.1186/1742-4690-12-S1-O22

**Published:** 2015-08-28

**Authors:** Toshie Nata, Juan Carlos Zapata, Raya Massoud, Steve Jacobson, Nazli Azimi, Yutaka Tagaya

**Affiliations:** 1 Cell Biology Lab, Institute of Human Virology, University of Maryland School of Medicine, Baltimore, MD, USA; 2 Viral Immunology Section, National Institutes of Neuronal Diseases and Stroke, Bethesda, MD, USA; 3 BIONIZ Inc., Irvine CA, USA

## 

We previously showed that the pathogenic mechanism of HAM-TSP involves the by-stander inflammatory damages of CNS cells by the hyper T-cell immunity caused by the HTLV-1. Thus we propose that suppressing this T-cell hyper-activation would be a key to prevent the progress of myelopathy in HAM-TSP. So far, three gc-family cytokines, IL-2, IL-15, and most likely IL-9 have been implicated in this process. Because these 3 cytokine share gc-subunit for signal transduction, they show functional redundancy. Because cellular responses to cytokines are sigmoid in shape with respect to cytokine doses, neutralizing one factor in a combination of 3 cytokines would not linearly lead to 33% inhibition even though the 3 factors were equally present in the mixture. This suggests that the use of a single monoclonal antibody (mAb) against one factor in a mixture of redundant cytokines often results in non-satisfactory effect, implying that a combinatorial blocking of all involved factors is needed to effectively treat clinical cases. A combined use of antibodies for each factor may solve the problem, but is infeasible in the clinic due to the costly nature of the Ab therapy. We therefore designed a peptide based on the conserved and divergent nature of the 6 gc-cytokines at their D-helix that is involved in their interaction with the gc-subunit. After computer simulated docking and high-throughput biological screening, we synthesized three peptides with different target specificities. The lead peptide (named BNZ 132-1) blocks IL-2, IL-9, and IL-15 in vitro. PEG-conjugation of this peptide enabled stability in vivo (T1/2 over 80 h in primates and in rodents) and efficiently blocked the in vivo propagation of an IL-15 dependent CD8 T cell leukemia in mice that we have generated in the past. In addition, BNZ 132-1 efficiently and specifically blocked the ex vivo activation and proliferation of CD8 T cells isolated freshly from HAM-TSP patients. We have already completed in vitro proof-of-concept, in vivo efficacy, toxicology/pharmacokinetics/pharmacodynamics studies and have obtained a patent on this compound. We will soon apply for the IND approval of this peptide for treating HAM-TSP. The structural nature, various unique biological characteristics and the drug potential of this peptide will be discussed.

